# FFA4/GPR120: Pharmacology and Therapeutic Opportunities

**DOI:** 10.1016/j.tips.2017.06.006

**Published:** 2017-09

**Authors:** Graeme Milligan, Elisa Alvarez-Curto, Brian D. Hudson, Rudi Prihandoko, Andrew B. Tobin

**Affiliations:** 1 Centre for Translational Pharmacology, Institute of Molecular, Cell and Systems Biology, College of Medical, Veterinary and Life Sciences, University of Glasgow, Glasgow, G12 8QQ, UK

**Keywords:** G protein-coupled receptor, free fatty acid, diabetes, cancer, lung function inflammation

## Abstract

Free Fatty Acid receptor 4 (FFA4), also known as GPR120, is a G-protein-coupled receptor (GPCR) responsive to long-chain fatty acids that is attracting considerable attention as a potential novel therapeutic target for the treatment of type 2 diabetes mellitus (T2DM). Although no clinical studies have yet been initiated to assess efficacy in this indication, a significant number of primary publications and patents have highlighted the ability of agonists with potency at FFA4 to improve glucose disposition and enhance insulin sensitivity in animal models. However, the distribution pattern of the receptor suggests that targeting FFA4 may also be useful in other conditions, ranging from cancer to lung function. Here, we discuss and contextualise the basis for these ideas and the results to support these conclusions.

## Trends

Substantial focus on the therapeutic potential of FFA4/GPR120 is currently directed towards type 2 diabetes.

Progress in the identification and characterisation of FFA4/GPR120 agonist ligands is apparent in both the primary scientific and patent literatures.

In models of glucose handling, FFA4/GPR120 agonists appear highly effective.

Recent indications provide support for consideration of FFA4/GPR120 ligands in areas of cancer treatment.

High levels of expression of FFA4/GPR120 in the lung suggest utility in analysis of the potential therapeutic roles of FFA4/GPR120 ligands in both acute and chronic airway inflammatory conditions.

## Receptors for Long-Chain Fatty Acids

In recent years, it has become clear that many components of foodstuffs, or metabolites derived thereof, act as homeostatic monitors of nutrient availability [1]. In many cases, such metabolites do so by binding to, and activating, members of the rhodopsin-like or ‘class A’ family of GPCRs [1]. Among such metabolites is the group of long-chain, nonesterified or ‘free’ fatty acids. A broad range of long-chain fatty acids of varying chain length and position and extent of unsaturation (Box 1) are able to activate a pair of these GPCRs 2, 3. Initially designated GPR40 [4] and GPR120 [5], upon acceptance that long-chain FFAs are indeed the key endogenous activators of these receptors, they were systematically renamed FFA1 (GPR40) [6] and FFA4 (GPR120) [7], although the initial, colloquial terminologies remain in widespread use. FFA1 has been validated clinically as a therapeutic target able to control blood glucose levels. Although the potential for FFA4 to also be a therapeutic target for regulating blood glucose levels and improving tissue insulin sensitivity appears clear from rodent model studies, this remains to be addressed in a clinical setting. However, recent studies exploring further roles of FFA4 in lung function and in the development of resistance to platinum-containing chemotherapeutics suggest that interest in FFA4 should be expanded beyond metabolic diseases and should also consider potential therapeutic applications of FFA4 antagonists as well as agonists.

Box 1 **Fatty Acids**

A fatty acid is a carboxylic acid with a linked aliphatic chain and may be either saturated or unsaturated. Most naturally occurring fatty acids contain a linear, unbranched aliphatic chain that lacks further modifications. However, in recent times, several modified forms, including hydroxy fatty acids [77] and branched fatty acid esters of hydroxy fatty acids 78, 79, have been suggested to have important biological roles via the activation of fatty acid-responsive GPCRs. Although both short-chain (C2–C5) and medium-chain (C9–C11)-length fatty acids are also known to activate specific GPCRs (short-chain fatty acids, FFA2, FFA3; medium chain fatty acids, GPR84), the longer-chain fatty acids activate both FFA1 and FFA4. While most long-chain fatty acids can be synthesised by the body, a pair of unsaturated long-chain fatty acids, α-linolenic acid [18:3(n-3)] and linoleic acid [18:2(n-6)] cannot, due to the lack of an appropriate desaturase enzyme. Therefore, these are defined as being ‘essential’ fatty acids. While both α-linolenic acid and linoleic acid contain 18 carbon atoms, they differ in the number of unsaturated bonds within the aliphatic chain (α-linolenic acid has three, whereas linoleic acid has two). Given that different fatty acids vary in chain length, to allow consistency of nomenclature the terminal carbon atom is designated ‘omega’ (n), after the last letter in the Greek alphabet. An ‘omega-3’ fatty acid has the first position of unsaturation three carbon atoms from the tail, while,for an ‘omega-6’ fatty acid, this is located six carbon atoms from the tail. As such, α-linolenic acid is coded as ‘18:3(n-3)’, while linoleic acid is coded as ‘18:2(n-6)’. Omega-3 fatty acids have attracted considerable attention as being beneficial for health, not least due to the high levels of such fatty acids in ‘oily’ fish, including mackerel and salmon. Before systematic redesignation as FFA4, in some publications this receptor (FFA4/GPR120) was highlighted as a (the) receptor for omega-3 fatty acids [80] and, although such fatty acids are among the most potent at this receptor, other long-chain saturated and unsaturated fatty acids are also able to activate this receptor [3].

## FFA1

FFA1 is highly expressed by pancreatic β cells and, because fatty acids are known, at least in acute settings, to promote insulin release from pancreatic islets, synthetic small-molecule activators of FFA1 were initially assessed for their ability to mimic this effect. Rapid translation to show that such ligands were also efficacious in various glucose tolerance tests, resulted in the optimisation of potentially drug-like FFA1 agonist ligands and the introduction into first-in-human clinical trials of fasiglifam {TAK-875, 2-[(3S)-6-({3-[2,6-dimethyl-4-(3-methylsulfonylpropoxy)phenyl]phenyl}methoxy)-2,3-dihydro-1-benzofuran-3-yl]acetic acid} 8, 9, 10 as a potential antidiabetic medication. Although substantial efficacy was noted in both Phase II and initial Phase III studies, development of fasiglifam was discontinued in late 2013 based on concerns of potential liver toxicity [11]. Subsequent reports have indicated that these adverse effects are likely related to the build-up of high concentrations of fasiglifam and an acyl glucuronide derivative of the ligand in the bile, reflecting blockage of various bile acid transporters including, but not limited to, the bile salt export pump (BSEP) 12, 13. Given the clinical validation of targeting FFA1, there remains significant interest in the potential of novel ligands at this receptor for the treatment of T2DM 14, 15, 16. Recent publications from various pharmaceutical companies several, of what appear, at least in animal models, to be highly effective and potent FFA1 ligands 17, 18, 19, support this. Clearly, issues akin to those that resulted in the removal of fasiglifam from clinical development, including inhibition of BSEP, would need to be addressed directly before further clinical studies commence.

## FFA4

GPR120 was deorphanised as the second receptor for long-chain fatty acids in 2005 [5]. Initial focus highlighted expression in the lower gut, the capacity of unsaturated fatty acids to promote release of the incretin glucagon-like peptide-1 (GLP-1) from the enteroendocrine cell line STC-1, and that both fatty acid-mediated elevation of phosphorylated extracellular regulated kinase (ERK) 1/2 MAP kinases and internalisation of the receptor from the surface of transfected cells could be used effectively as means to screen for, and identify, synthetic agonists at the receptor [5]. These initial studies also highlighted the importance of the carboxylate of the fatty acids to their function because equivalent methyl esters were inactive. Although the authors also reported [5] the ability of α-linolenic acid [18:3(n-3); Box 1] to increase levels of both GLP-1 and insulin in both the portal vein and inferior vena cava of mice, there remains uncertainty over the contribution of such released GLP-1 to the observed increase in circulating insulin levels and whether this is also the case in humans. Of particular interest, although not explored further in the initial studies, was the observation of particularly high levels of receptor mRNA in the lung of both humans and mice [5]. The potential relevance of this is discussed below. Although only distantly related in terms of sequence to FFA1, acceptance of GPR120 as a *bona fide* GPCR responsive to long-chain fatty acids resulted in its systematic re-classification as FFA4 [7].

Although detailed and comprehensive analyses have shown that a broad swathe of saturated as well as unsaturated fatty acids are able to activate FFA4 [20] (reviewed in [3]), initial description of the capacity of α-linolenic acid to activate this receptor paved the way for a particular focus on the effects of this and other health-beneficial omega-3 fatty acids acting either predominantly, or even exclusively, via FFA4 21, 22, 23, 24, 25, 26, 27, 28. This is undoubtedly an oversimplification. For example, there have been suggestions that several beneficial effects of omega-3 fatty acids do not require FFA4 29, 30, 31, 32, 33. However, it is important to note equivalent studies where using combinations of FFA4 expression knockout and animals that synthesise high levels of polyunsaturated omega-3 fatty acids, resulted in the suggestion that such fatty acids regulate beneficially vascular inflammation and neointimal hyperplasia via FFA4 [34]. A challenge for the translation of these ideas to humans is whether, even with dietary supplementation, levels of such fatty acids are likely sufficient to engage the receptor to a substantial level [35]. This question is further complicated by the fact that quantitatively more prevalent fatty acids are also able to activate FFA4 [20], while specific, but relatively uncommon, fatty acids or their derivatives, which do not display substantially greater potency at FFA4 *in vitro*, are capable of generating biological functions with apparent high potency and that are lacking in FFA4-knockout animals [36] or are reduced with knock down of this receptor in model cell systems [37].

## Signalling Mechanisms of FFA4

**Figure 1:**
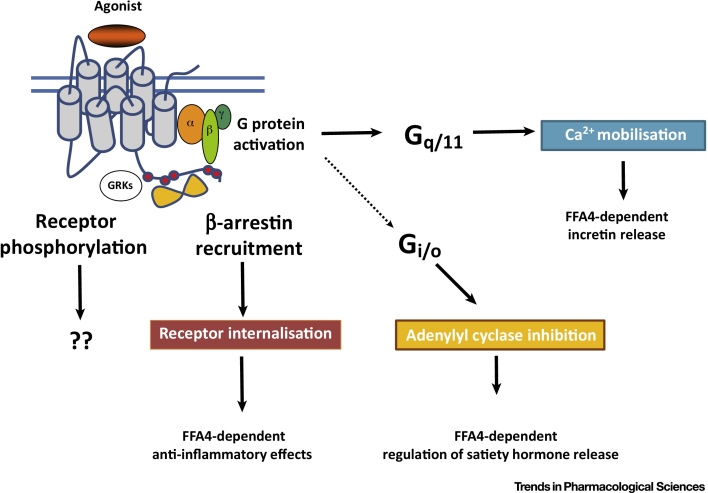
Free Fatty Acid Receptor 4 (FFA4) Can Engage Multiple Signalling Pathways to Regulate Distinct Physiological Outcomes. Agonist-induced interactions with members of the G_q_/G_11_ G protein family, resulting in elevated intracellular [Ca^2+^], are observed following expression of FFA4 in heterologous cell systems. This pathway is also central to many effects of FFA4 in a physiological context [2]. However, release of the satiety hormone ghrelin is sensitive to treatment with pertussis toxin [42], defining a role for G_i_-family G proteins in this endpoint. Many efforts to identify synthetic agonists at FFA4 have used receptor-β-arrestin interaction assays, and key physiological roles for such β-arrestin-mediated interactions include regulation of production of anti-inflammatory mediators from macrophages [21]. Although often interlinked, effects defined by agonist-induced phosphorylation of FFA4 can be resolved from effects generated following FFA4 with arrestin [41]. Further work, potentially involving the use of novel transgenic mouse lines expressing phosphorylation-resistant (see Figure 2 in the main text) variants of FFA4 are likely to help unravel distinct roles of these regulatory interactions.

**Figure 2:**
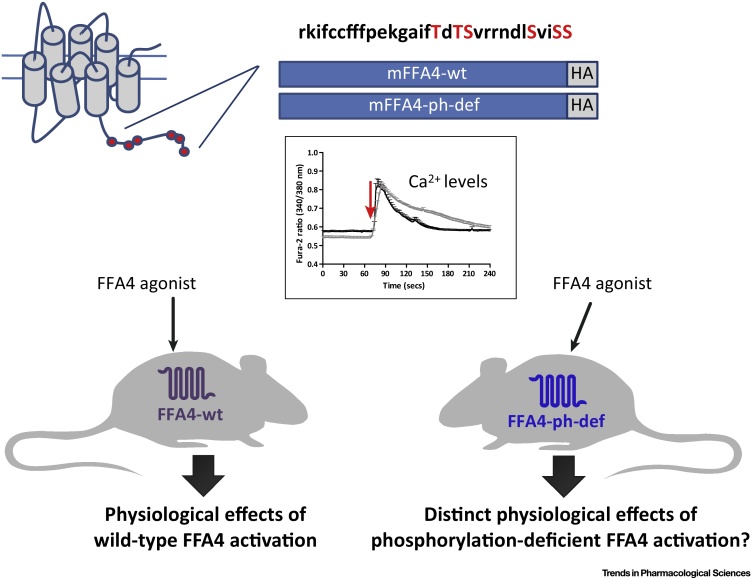
Regulation of the Phosphorylation State of Free Fatty Acid Receptor 4 (FFA4): Potential Physiological Roles. Both mouse (m) [48], as illustrated here (red circles in the cartoon of the seven transmembrane domain organisation of the receptor structure, and red font in the sequence of specific amino acids within the indicated region of its primary amino acid sequence) and human [47] FFA4 become rapidly phosphorylated on two groups of Ser/Thr residues within the intracellular C-terminal tail of the receptor upon addition of an agonist ligand. Alteration to generate the phosphorylation-defective form (ph-def) results in extended maintenance of elevated Ca^2+^ levels when either wild-type mouse FFA4 (wt; black) or ph-def mouse FFA4 (grey) are expressed in HEK293 cells and exposed to an agonist ligand (red arrow). Generation of transgenic mice in which wild-type FFA4 is replaced with either a C-terminally HA epitope-tagged form of FFA4 (left-hand side) or a similarly epitope-tagged form of ph-def FFA4 (right-hand side) is allowing analysis of receptor expression patterns (see Figure 4 in the main text) and specific roles of agonist-mediated phosphorylation of FFA4. Studies using these animals are still to be reported in the primary literature.

The ability of FFA4 to elevate intracellular levels of Ca^2+^ 5, 38, 39, 40 provided early predictions of a key role of the phosphoinositidase C-linked G proteins G_q_ and/or G_11_ in transduction of signals from this receptor, while later studies that examined production of inositol phosphates [41] provided further support. The importance of this group of G proteins to key aspects of FFA4 function has been confirmed by the ability of selective G_q_/G_11_ inhibitors to block such signals [41]. When expressed in HEK293 cells that had been genome-edited to lack expression of both G_q_ and G_11,_ FFA4 was unable to induce elevation of either inositol phosphates or intracellular Ca^2+^ levels [41]. Although this appears to be the dominant mode of G protein-mediated signalling for FFA4 (Figure 1), several reports suggest that treatment with pertussis toxin eliminates the ability of this receptor to regulate the release of the satiety hormone ghrelin [42] (Figure 1) and also the release of somatostatin from delta cells of the pancreas [43]. This indicates a key role for G_i_-family G proteins in these processes. Segerstolpe *et al.* [44] noted expression of mRNA encoding FFA4 in delta cells isolated from both healthy individuals and those with T2DM that was higher than expression of this receptor in pancreatic β cells derived from the same individuals. Interestingly, and by contrast, FFA4 mRNA expression was not detected in either α or γ cells [44]. This expression profile in cell subtypes may have substantial significance and could be exploited if FFA4 agonists can be identified that show ‘bias’ between promoting signalling via G_q/11_ and G_i_-family G proteins. In initial deorphanisation studies, Hirasawa *et al.* [5] used measures of the internalisation of FFA4 from the surface of transfected cells. Such agonist-induced internalisation of FFA4 is both robust and extensive 39, 40 and, at least in the context of HEK293 cells, is almost entirely dependent on interactions with an arrestin adapter protein [41] (Figure 1). Non-canonical, non-G-protein-mediated, signalling role(s) of such a FFA4/arrestin complex remain to be fully defined. For example, although arrestins are often linked to aspects of the temporal profile of GPCR-mediated regulation of the ERK1/2 MAP kinases, Alvarez-Curto *et al.* [41] did not identify a substantial role for arrestins in FFA4-mediated phosphorylation of ERK1/2 when using HEK293 cells genome-edited to lack expression of either Gα_q_ plus Gα_11_, or of β-arrestin 1 + β-arrestin 2. Moreover, the key role of arrestins in FFA4 signalling in these cell backgrounds was their more traditional role in acting to desensitise G protein-mediated signalling because their elimination resulted in Ca^2+^ ‘spikes’ being generated repetitively with maintained exposure to an agonist [41]. Despite this, the now well-established anti-inflammatory roles of FFA4 expressed within immune cell populations where, in mice, particularly high levels are reported in thymus CD8^+^ dendritic cells and in lung-resident macrophages (Immunological Genome Projecti) has focused attention on potential contributions of interactions of FFA4-associated arrestins with the transforming growth factor beta (TGF-β)-activated kinase 1 binding protein 1 (TAB-1). This is believed to limit TAB-1 interacting with TGF-β-activated kinase 1 (TAK1), a complex that is important for transmitting signals from activated cell surface Toll-like receptors (TLRs), and the receptor for tumour necrosis factor alpha (TNFα), for the production and release of proinflammatory mediators [45]. The sustained interaction between agonist-occupied FFA4 and an arrestin is based on agonist-promoted phosphorylation of several serine and threonine residues located in the intracellular, C terminal of the receptor 46, 47, 48 (Figure 2). Conversion of these residues to non-hydroxyl amino acids greatly reduces such interactions, while production of antisera that recognise amino acids within the C-terminal region specifically only when they are phosphorylated 41, 47, 48 has provided reagents able to assess the state of receptor activation. In humans, splice variation within the third intracellular loop of FFA4 produces a ‘long’ isoform containing 16 additional amino acids [49]. Although expression of the long variant seems to be restricted, it appears to act as a ‘biased’ receptor, unable to engage with G protein-mediated signalling systems, but is able to interact with β-arrestins as the short isoform and to undergo internalisation in an agonist-dependent manner [39]. The broader implications of this remain unclear because there is no obvious tissue situation in which the long isoform is uniquely expressed and in which only β-arrestin- or non-G protein-mediated signalling might therefore be anticipated.

## Synthetic Agonists for FFA4

Long-chain fatty acids can be rapidly converted to different biologically active species that function at GPCRs other than FFA1 and/or FFA4. Given that such interconversion is challenging to limit *in vivo*, direct studies with fatty acids can be difficult to interpret unequivocally. As such, the identification and characterisation of synthetic ligands with affinity for FFA4 have been integral to better understanding the biological functions of this receptor (Table 1). However, owing to the strongly overlapping profile of fatty acids as activators of FFA4 and FFA1, many of the initially described synthetic ligands with activity at FFA4 are also able to activate FFA1 (Table 1). This reflects their structural similarity to fatty acids. More recently described ligands display improved selectivity for FFA4 over FFA1 (Table 1).

**Table 1:** Structures of Key Ligands with Activity at FFA4

Ligand	Structure	Mode of action	Selectivity	Other comments	Refs
TAK-875		Agonist	FFA1		[11]
GW9508		Agonist	FFA1 >>FFA4		38, 21
NCG21		Agonist	FFA1 = FFA4		50, 51
TUG-891		Agonist	FFA4 > FFA1	Degree of selectivity is species dependent	52, 40
GSK137647A		Agonist	FFA4		[56]
TUG-1197		Agonist	FFA4		[57]
AH-7614		Antagonist	FFA4		56, 60
TUG-1387		Inactive	–		[60]

The first described synthetic FFA1 active agonist, GW9508 (4-{[(3-phenoxyphenyl)methyl]amino}benzenepropanoic acid), was immediately shown to also activate FFA4, although with some 100-fold lower potency [38]. Therefore, in the initial absence of FFA4-selective synthetic agonist ligands, GW9508 was used as a FFA4 agonist in several studies using cells and tissues that lacked detectable levels of co-expressed FFA1 (e.g., [21]). Probably because this ligand is available commercially, this approach has continued (reviewed in [2]). Early efforts to produce FFA4-selective ligands reported only modest success. For example, Suzuki *et al.* [50] modified PPARγ-active molecules to generate a ligand, 4-{4-[2-(phenyl-pyridin-2-yl-amino)-ethoxy]-phenyl}-butyric acid, later named NCG21 (Table 1), which displayed modest selectivity for FFA4 over FFA1, but also only modest potency [51]. With appropriate recognition that it probably acts as a combined FFA4 and FFA1 activator, this compound has been used recently alongside other more-selective ligands to help unravel the contribution of each long-chain fatty acid receptor to the ability of the omega-3 fatty acid, hexadeca-4,7,10,13-tetraenoic acid [16:4(n-3)], to generate systemic resistance to the DNA-damaging chemotherapeutic cisplatin [36] (discussed below). In the first significant advance in developing FFA4-selective agonist ligands, Shimpukade *et al.* [52] reported the *ortho*-biphenyl ligand 4-{[4-fluoro-4′-methyl(1,1′-biphenyl)-2-yl]methoxy}-benzenepropanoic acid (TUG-891) (Table 1). This molecule showed good potency at both human and mouse FFA4 and 1000-fold selectivity over human FFA1 in assays based on the induced interactions between the receptor and β-arrestin 2. These studies also reported loss of agonist function of TUG-891 at an Arg^99^ to Gln mutant of human FFA4 and, alongside the parallel mutagenesis studies of Watson *et al.* [39], provided the first direct evidence of the key role of this arginine residue in coordinating the carboxylate of fatty acids and fatty acid-like synthetic ligands.

GPCRs responsive to fatty acids have been shown to display substantial variation in pharmacology between species orthologues [53]. More extensive studies with TUG-891 illustrated that selectivity reported between human FFA4 and FFA1 was significantly less pronounced at mouse orthologues [40] and also varied when measuring G protein-mediated Ca^2+^ elevation versus receptor interactions with an arrestin [40]. Although there is no comprehensive analysis of these features for many ligands, which are rarely reported beyond humans versus mice, the major reason for the lower selectivity between the mouse orthologues is their higher potency at mouse FFA1 rather than reduced potency at mouse FFA4. In practise, although a potent and selective FFA4 agonist in human cells and tissues, TUG-891 is best described as a dual FFA4 and FFA1 agonist in equivalent tissues from rodents [36]. More recently reported compounds have provided greater levels of selectivity. For example, Adams *et al.* [54] reported a chromane propionic acid-based agonist series where specific members are at least 300-fold more selective for both human and mouse FFA4 compared with FFA1. Similarly, Sparks *et al.* [55] described a phenylpropanoic acid series with an exemplar compound showing between 40- and 130-fold selectivity over FFA1 across human, mouse, and rat orthologues.

Similar to free fatty acids, all of the compounds described above contain a carboxylate that has been shown directly (or at least modelled) to interact with Arg^99^ of FFA4. However, a pair of recent reports has also described sulfonamide-containing FFA4 agonists 56, 57. GSK137647A [4-methoxy-*N*-(2,4,6-trimethylphenyl)benzenesulfonamide] (Table 1) is reported to have greater than 50-fold selectivity for FFA4 over FFA1 and that this is preserved across species [56]. Similarly, a potent nonacidic sulfonamide FFA4 agonist, TUG-1197 {2-[3-(pyridin-2-yloxy)phenyl]-2,3-dihydrobenzo[*d*]isothiazole 1,1-dioxide} (Table 1) is described as having no detectable activity at FFA1 [57]. Despite the nonacidic nature of the compound, both mutational and modelling studies indicated that it likely binds within the same orthosteric binding pocket as the carboxylate-containing agonists that resemble synthetic fatty acids [57]. Clearly, the more selective nature of several recently disclosed ligands offers potential for more defined analysis of FFA4-mediated functions, and several such ligands (e.g., GSK137647A) are now available from commercial sources. However, not all of the more recently described ligands are suitable for *in vivo* studies due to poor pharmacokinetic and pharmacodynamic properties [56]. By contrast, although not currently available from commercial suppliers, phenylpropanoic acid ‘compound 29’ [55], nonacidic sulfonamide ‘compound 34’ [57], and chromane propionic acid ‘compound 18’ [54] have each been used for rodent *in vivo* studies to explore glucose handing and aspects of regulation of insulin sensitivity. In each case, these have provided clear support for an important role of FFA4 in the regulation of glucose homeostasis. The emergence of chemically distinct series of FFA4 agonists allows the possibility of using pairs of compounds from different series to provide greater support for specific roles of FFA4 [58]. Even if the full off-target profile of each ligand is not currently available, it is reasonable to assume that compounds derived from different chemotypes will produce varying non-FFA4-mediated effects. Although no FFA4 agonist has yet entered clinical studies, there is considerable expectation that such ligands may offer novel combinations of benefits in T2DM 53, 59.

## Synthetic Antagonists for FFA4

To date, only compounds from a single chemical series have been reported as FFA4 ‘antagonists’ (Table 1). ‘Compound 39’ (4-methyl-*N*-9*H*-xanthen-9-yl-benzenesulfonamide), now available from commercial vendors as AH-7614, was initially reported as an antagonist at this receptor [56]. This compound, and a closely related molecule, 4-methyl-*N*-(9*H*-thioxanthen-9-yl)benzenesulfonamide (TUG-1506) [60] (Table 1), act as noncompetitive, negative allosteric modulators of the action of a range of FFA4 agonist chemotypes [60]. Although neither competitive nor displaying more than moderate affinity (approximately 10 nM) [60] for the receptor, AH-7614 has recently been used in a range of studies; for example, to confirm a specific role for FFA4 as the long-chain fatty acid receptor on splenic macrophages responsible for release of a lysophosphatidic acid species that is able to produce systemic resistance to platinum-containing chemotherapeutics [36]; to identify a role of the receptor in activation of brown fat [61]; to explore whether various effects of arachidonic acid are produced via FFA4 [62]; and the contribution of FFA4 to docosahexaenoic acid [20:6(n-3)]-mediated effects in GnRH-producing neurones [63]. However, as noted by Watterson *et al.* [60], AH-7614 is a simple xanthene-containing chemical and, although this molecule does not block agonist effects at FFA1 [60], other potential sites of action have not been explored. Therefore, it is noteworthy that, in studies in rat round spermatids, AH-7614 itself induced an increase in [Ca^2+^]_i_ in the absence of FFA4 receptor-activating ligands [64], akin to those produced by the omega-6 fatty acid arachidonic acid [20:4(n–6)]. Given such concerns, Watterson *et al.* [60] suggested as a control the parallel use of 4-methyl-*N*-(9*H*-xanthen-9-yl)benzamide (TUG-1387) (Table 1), an analogue of both AH-7614 and TUG-1506 that has no activity at FFA4. Whereas AH-7614 blocked autocrine differentiation of mouse preadipocytes towards an adipocyte phenotype, TUG-1387 did not [60], providing stronger evidence for a direct role of FFA4 in this process.

## Therapeutic Opportunities for FFA4 in Cancer

**Figure 3:**
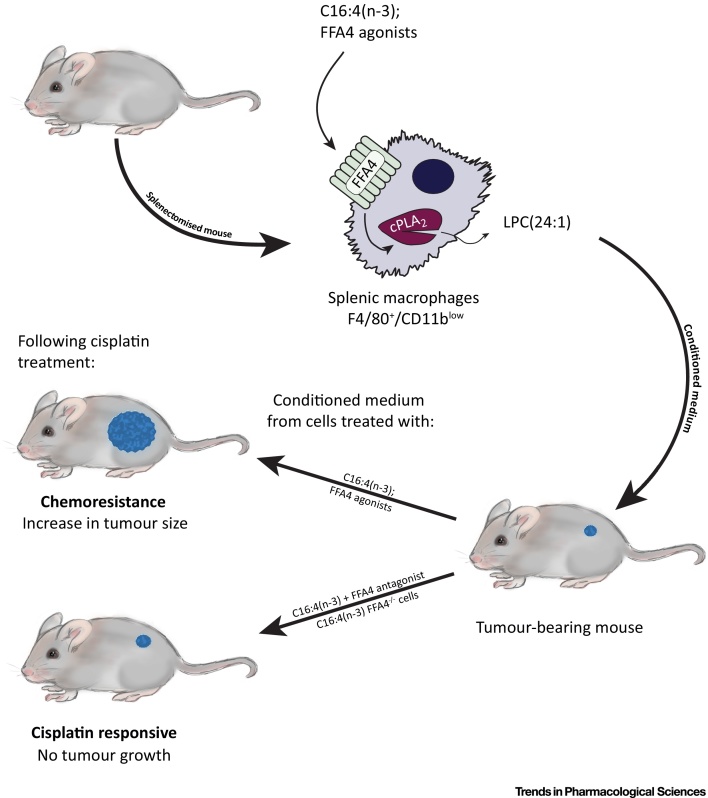
A Role for Free Fatty Acid Receptor 4 (FFA4) in Mediating Resistance to Platinum-Containing Chemotherapeutics. Cisplatin and other platinum-based therapeutics are integral to chemotherapy. However, the development of resistance to such agents limits their effectiveness. In a mouse model, the fatty acid 16:4(n-3) was identified to contribute to the development of resistance in a manner linked to a key subpopulation of splenocytes. 16:4(n-3) is able to activate both FFA4 and FFA1, and these two G-protein-coupled receptors are co-expressed by this splenocyte population. Medium ‘conditioned’ by exposure of splenocytes to 16:4(n-3) is able to induce resistance to cisplatin when injected into tumour-bearing mice and both pharmacological and receptor ‘knockout’ studies demonstrated the effect of 16:4(n-3) to be mediated specifically by FFA4 [36]. The mechanism was shown to involve FFA4-mediated activation of splenocyte cytosolic (c)PLA_2_, resulting in the release of a specific lysophosphatidic acid species C24:1, with this isolated lipid species able to mimic the effect of conditioned medium [36].

Beyond T2DM, the therapeutic area in which FFA4 has perhaps attracted greatest attention to date is cancer. In part, this reflects appreciated roles for fatty acids and fat-rich diets in either promoting cancer cell growth and motility or the capacity of health-beneficial fatty acids, including omega-3 fatty acids, to reduce the growth of several types of tumour. The significance of a number of the reported studies is hard to define, in that many have focused largely on the effects of fatty acids alone and have frequently suggested potential contributions of both FFA4 and FFA1. Several of these studies have recently been reviewed elsewhere [65]. However, growing appreciation of the developing pharmacology at FFA4 and at FFA1 has provided new insights into the potential for FFA4 ligands. A key example is the contribution of FFA4 to the development of systemic resistance to cisplatin-based chemotherapy. Mesenchymal stem cells produce a pair of polyunsaturated fatty acids [66], 12-*S*-hydroxy-5,8,10-heptadecatrienoic acid (12-*S*-HHT), an activator of the leukotriene B4 receptor 2 [67], and hexadeca-4,7,10,13-tetraenoic acid [16:4(n-3)], which, similar to many other fatty acids, can activate both FFA4 and FFA1 with similar potency [36], at least *in vitro*. Recent studies used a combination of the genetic elimination of FFA4 in mice and a range of both markedly selective and dual-acting FFA4 and FFA1 agonists, in concert with selective antagonists of each receptor, to show that, although both FFA4 and FFA1 are expressed by a key population of splenic macrophages, activation by 16:4(n-3) of FFA4 on these cells specifically induced a signalling cascade that, via activation of cytosolic phospholipase A2, resulted in the release of several species of lysophosphatidic acid into the medium (Figure 3). Such ‘conditioned medium’ was able to induce resistance to cisplatin-induced DNA damage in tumour cells and, subsequently, to limit the effectiveness of cisplatin to inhibit tumour growth when injected into tumour-bearing mice [36]. Moreover, a single lysophosphatidic acid species, C24:1, was able to replicate the effect of 16:4(n-3)-conditioned medium, suggesting C24:1 as a likely end-effector, although the molecular basis for the effect of lysophosphatidic acid C24:1 remains undefined [36]. Addition of either dual-acting FFA4/FFA1 agonists, including TUG-891 [52] and NCG21 [51], but, more importantly, also the highly selective FFA4 agonist TUG-1197 [57], to splenic macrophages isolated from wild-type mice generated conditioned medium that was as effective in producing chemoresistance as treatment with 16:4(n-3) [36]. By contrast, addition of 16:4(n-3) to splenic macrophages isolated from mice lacking expression of FFA4 did not generate conditioned medium that was able to replicate this effect. Moreover, co-addition of the FFA4 antagonist AH-7614 blocked the ‘conditioning’ effect of 16:4(n-3) in cells isolated from wild-type animals [36]. Together, these results suggest the potential of FFA4 antagonists to either limit the development of resistance to platinum-containing chemotherapeutic agents or to spare the dose of such agents required for efficacy. However, the effect of the second platinum-induced fatty acid, 12-S-HHT, was not lost in cells isolated from FFA4 knockout mice [36]. As such, blockade of FFA4 with a synthetic antagonist is unlikely to be fully effective *in vivo* if used in isolation.

As well as the above studies, Meier and coworkers used TUG-891, alongside omega-3 fatty acids, to show a potential role for FFA4 in inhibiting proliferation of DU145 prostate cancer cells [68]. Given that these cells express both FFA4 and FFA1 and the current view that TUG-891 may not be sufficiently selective to fully differentiate between the two fatty acid receptors, the fact that FFA4 knockdown prevented TUG-891-induced inhibition of growth and migration provided extra support for a key role of FFA4 [68]. However, the obvious conclusion from these studies is that FFA4 agonism, rather than antagonism, as suggested in limiting the development of induced chemoresistance to cisplatin treatment, might be effective in this context. In a subsequent study, the same group used a pair of FFA1/FFA4-active agonists to examine possible roles of these GPCRs in the proliferation of a pair of breast cancer cell lines. However, although the pharmacological studies were unable to clearly discriminate between the effects of the ligands as reflecting activation of FFA1 or FFA4, both the MCF-7 and MDA-MD-231 cell lines appeared to express significantly higher levels of mRNA encoding FFA1 than FFA4. Moreover, although immunoblotting studies potentially detected two variants of FFA4 in MCF-7cells, equivalent forms were lacking in MDA-MD-231 cells. Based on these observations, the authors concluded that the major contributions of GW9508 and TUG-891 were likely to be mediated via FFA1 [69].

## Other Potential Therapeutic Opportunities in Targeting FFA4

**Figure 4:**
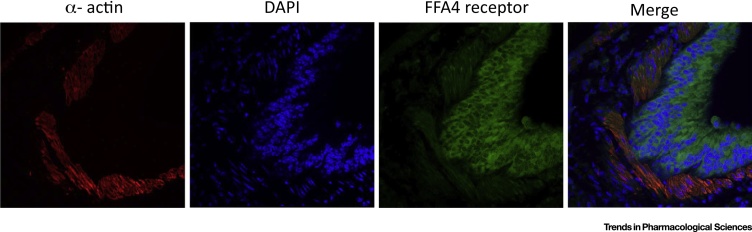
Immunohistochemical Detection of Free Fatty Acid receptor 4 (FFA4) in Mouse Lung. Antigen-retrieved, formalin-fixed paraffin-embedded lung tissue slices obtained from wild-type mice were incubated with anti-FFA4 antibodies [48]. They were incubated subsequently with Alexa 488-conjugated secondary antibody (green) and cy3-conjugated anti α-actin (to detect smooth muscle) (red). Following further washes, samples were placed on coverslips with a mounting medium containing the nuclear stain DAPI (blue). Images were taken using a confocal microscope. FFA4 appears to be expressed predominantly within the epithelial layer.

Both mRNA expression patterns and availability of selective FFA4 antibodies have revealed that this receptor is expressed in a variety of tissues, pointing to a range of functions yet to be fully defined [5]. For example, FFA4 was found to be highly expressed in murine lung [70] (Figure 4), where clues to its function are only just beginning to emerge. Expression in this organ appears restricted to the airway epithelium [70], which primarily comprises mucous-secreting goblet cells and ciliate columnar epithelial cells. The role of FFA4 in these various cell types is unknown, but it is of interest that the dietary-derived omega-3 fatty acids, docosahexaenoic acid [22:6(n-3)] and eicosapentaenoic acid [20:5(n-3)], have been reported to be enriched in airway mucosa [71], suggesting that there is a ‘store’ of endogenous ligands for the FFA4 receptor located at the lung epithelium. Furthermore, a recent study suggested that FFA4, acting on epithelial club cells, promotes bronchial epithelial repair following naphthalene-induced epithelial injury [72]. This study may provide a potential explanation for the observed benefits of clinical administration of omega-3 fatty acid-rich fish oils in human lung injury [73]. Further studies on the role of FFA4 in lung function are clearly warranted.

Studies on the possibility that FFA4 regulates central functions are similarly in their infancy. However, FFA4 immunoreactive neurones have been identified in the hypothalamus, where they are thought to protect against hypothalamic dysfunction in obesity [74]. The mechanism appears to have two distinct components. The first is a reduction in hypothalamic inflammation mediated by downregulation of TLR4 and TNF-induced inflammatory pathways [74]. The second is via neuropeptide Y-expressing hypothalamic neurones that co-express FFA4, through which omega-3 fatty acids are seen to reverse obesity-induced resistance to leptin [74]. These central FFA4-mediated mechanisms to regulate food intake and body mass likely work in concert with peripheral pathways, as exemplified by studies on the release of ghrelin, a key hormone that mediates food-seeking behaviour and food intake as well as adiposity. Dietary-derived long-chain fatty acids targeting FFA4 on ghrelin-expressing cells act to inhibit the secretion of ghrelin [75], thereby providing a negative feedback loop to reduce food intake.

## Concluding Remarks

Although potential therapeutic opportunities from targeting FFA4 are currently focussed firmly on T2DM and other metabolic indications, including nonalcoholic steatohepatitis, the broader expression pattern of the receptor suggests wider roles in (patho)physiology. Full understanding of the repertoire of physiological roles of FFA4 has been restricted by the focus on T2DM and the beguiling prospect that it may result in an entirely novel therapeutic entity. However, the continuing development of tool compounds, vital for detailed pharmacological analysis, in concert with the use of novel genetically engineered mice where *in vivo* FFA4 signalling is modulated (Figure 2), as used for other GPCRs (e.g., the muscarinic acetylcholine M_3_ receptor [76]), is likely to highlight further opportunities (see Outstanding Questions). Two opportunities that are beginning to emerge are the role of FFA4 in cancer and the possibility that the high level of expression of FFA4 in lung will be linked to key physiological endpoints associated with airway function and dysfunction.

## Conflict of Interest

Both GM and BDH are shareholders in Caldan Therapeutics, a company exploring potential novel treatments for type 2 diabetes.

Outstanding Questions Will FFA4 become a validated therapeutic target in T2DM? Although FFA4 is considered a candidate therapeutic target for T2DM, to date no clinical studies have been initiated. Key outstanding questions include whether agonist ligands with appropriate drug-like characteristics can be identified, whether these will provide features and outcomes above and beyond glucose-lowering in rodent models and whether potential safety issues associated with clinically used FFA1/GPR40 agonists can be mitigated against or overcome.

How important will the anti-inflammatory effects of FFA4 be to clinical delivery? Although activation of FFA4 expressed by macrophages results in the regulation of proinflammatory mediator release via a mechanism reported to involve interaction of the receptor with an arrestin adaptor protein, the broader roles of both non-G protein-mediated signaling and of activation of G protein-dependent pathways other than those involving the phosphoinositidase C-linked G proteins G_q_/G_11_ remains to be fully elucidated and understood. Clear analysis of these aspects of FFA4 signaling will define whether a search for agonist ligands that display ‘bias’ might be useful.

Might FFA4 become a therapeutic target for treatment of nonalcoholic steatohepatitis? Although little direct information is yet available on this topic, considerable interest in this possibility has been voiced

Will FFA4 antagonists provide a mean to enhance the chemotherapeutic index of platinum-based chemotherapeutics? Recent studies suggest that ‘platinum’ chemotherapy induces the generation of the fatty acid hexadeca-4,7,10,13-tetraenoic acid and that, by activating FFA4/GPR120 expressed by a splenocyte subpopulation, this fatty acid promotes release of a isoform of lysophosphatidic acid that appears to act as the final mediator of the development of resistance to platinum-based chemotherapeutics. Defining the molecular target for this lysophosphatidic acid may be key in providing novel approaches to either limit the development of such chemoresistance or to allow the effective use of lower doses.

What therapeutic opportunities may be identified for regulation of FFA4 in airway function? Early studies of the distribution of FFA4/GPR120 highlighted that mRNA encoding the receptor was expressed abundantly in lung. Assessment of the role of FFA4/GPR120 in lung is currently at an early stage, but is likely to provide a rich source of novel insights.

## Resources

^i^www.immgen.org/databrowser/index.html
